# 3D Printing of New Foods Using Cellulose-Based Gels Obtained from *Cerotonia siliqua* L. Byproducts

**DOI:** 10.3390/gels10120759

**Published:** 2024-11-23

**Authors:** Antoni Capellà, Mónica Umaña, Esperanza Dalmau, Juan A. Cárcel, Antoni Femenia

**Affiliations:** 1 Department of Chemistry, University of the Balearic Islands, Ctra. Valldemossa, Km 7.5, 07122 Palma, Spain; antoni.capella@uib.cat (A.C.); monica.umana@uib.es (M.U.); esperanza.dalmau@uib.es (E.D.); 2 Analysis and Simulation of Agro-Food Processes Group, Universitary Institute of Food Engineering-FoodUPV, Universitat Politècnica de València, Camino de Vera S/N, 46022 Valencia, Spain; jcarcel@tal.upv.es

**Keywords:** carob byproduct, cellulose-rich fraction, 3D food printing, cellulose-based gel

## Abstract

Carob pulp is a valuable source of cellulose-rich fraction (CRF) for many food applications. This study aimed to obtain and characterize a CRF derived from carob pulp waste after sugar removal and to evaluate its potential use in the 3D printing of cellulose-rich foods. Thus, the extraction of the CRF present in carob pulp (by obtaining the alcohol-insoluble residue) was carried out, accounting for nearly 45% dm (dry matter) of this byproduct. The CRF contained about 24% dm of cellulose. The functional properties (swelling capacity, water retention, and fat adsorption) related to this fraction were determined, showing a value of 5.9 mL/g of CRF and 4.0 and 6.5 g/g of CRF, respectively. Different gels were formulated with a total solids content of 15% wm (wet matter), using potato peel flour as a base and partially substituting with CRF (0% to 8% wm). The cellulose-based gels were characterized in terms of viscosity, water distribution (low-field Nuclear Magnetic Resonance), and printability, while the 3D printed samples were assessed for their textural properties. As the percentage of added CRF increased, the viscosity decreased while the water retention increased. Printability improved when small proportions of CRF (2% to 4%) were used, while it deteriorated for higher percentages (6% to 8%). The textural properties (hardness, adhesiveness, cohesiveness, and gumminess) showed significant changes caused by the addition of CRF, with gels containing 3% to 4% CRF exhibiting the most suitable printing values. In summary, this study demonstrates the significant potential of carob cellulose-based gel as an ingredient in the 3D printing of novel fiber-rich foods, contributing to reducing food waste and promoting sustainable practices within the framework of the circular economy.

## 1. Introduction

Currently, food waste is a serious global problem, as it significantly contributes to greenhouse gas emissions. In fact, it is estimated that globally, food waste is responsible for approximately 6% of total greenhouse gas production. In addition to the pollution generated by this waste, there is a valuable loss of nutrients from unused food [1].

Among the different residues, lignocellulosic residues stand out because of their high polysaccharide content. Lignocellulosic biomass comes from different natural organic plant-based materials. The structure of the cell wall in this biomass consists mainly of three types of biopolymers: cellulose, hemicellulose, and lignin [2].

Cellulose, regardless of its source, is composed of D-glucopyranose ring units in the low-energy ^4^C_1_-chair conformation. These units are connected by β-1,4-glycosidic bonds, causing a 180° rotation of the chain axis. The repeating unit of cellulose is cellobiose, measuring 1.3 nm in length [3]. On the other hand, hemicelluloses show backbones of glucose, mannose, and/or xylose linked by β-(1→4) bonds, and they interlace with cellulose, proteins, and lignin mainly through noncovalent interactions such as hydrogen bonds. Lignin is an amorphous, crosslinked copolymer synthesized randomly from three different diphenylpropane monomers: p-coumaryl alcohol, coniferyl alcohol, and sinapyl alcohol. These monomers are synthesized via the phenylpropanoid pathway in plants and differ in their degree of methoxylation [4].

Carob pulp is an example of a lignocellulosic agrifood residue. The carob tree (*Ceratonia siliqua* L.) is a perennial tree that belongs to the Leguminosae or Fabaceae family (subfamily Caesalpinioideae) and has its origins in the Mediterranean region. Since ancient times, it has been widely used due to its edible fruits, known as carobs [5].

Carob seeds are recognized as the most valuable part of this fruit and are used by the industry to produce locust bean gum. It is a natural food additive (LBG, E410) widely used as a thickening and stabilizing agent. On the other hand, carob pulp, which is obtained after extracting the seeds, has been undervalued and considered an agricultural waste or with little commercial value.

Despite not being fully utilized and valued, several studies have demonstrated the remarkable health benefits that carob can provide. This fruit is notable for its content of soluble sugars, low fat levels, high polysaccharides content, and the presence of various bioactive molecules such as polyphenols [6]. Sugars, mainly sucrose, are usually extracted to produce carob syrup. After sugar extraction, this material is an important source of insoluble fiber, mainly composed of lignin, cellulose, and hemicellulose [7], comprising approximately 30 to 40% of the carob pulp [8].

Studies on carob pulp have demonstrated its potential as a source of dietary fiber and polysaccharides, such as cellulose, for improving the rheological properties of foods. For example, carob flour has been successfully integrated into bread formulations to enhance dough characteristics, improve texture, and promote moisture retention [9]. Its use in food products contributes to the overall sensory and functional properties, making carob pulp a valuable natural ingredient for enriching foods with fiber while optimizing their texture and stability [9]. Similarly, Restuccia et al. [10] observed that adding carob pulp to gluten free bakery product formulations led to lower cohesive interaction and rigidity.

Cellulose-rich solids can be used in the production of gel-type materials, which have several interesting applications [11]. For instance, gels produced from different types of cellulose have been used in the formulation of ink for 3D printing [12].

While 3D printing technology was initially mainly used in nonfood sectors, it is now being applied in the production of a wide range of foods. 3D printing in the food sector offers the opportunity to create personalized products, improve their nutritional content, enhance health benefits, and extend their shelf life [13]. Extrusion-based printing is the most widely used for 3D printing of foods. This type of printing is primarily achieved by the displacement of a piston that pushes a molten or semisolid material contained in a syringe through a cannula. Essentially, the printing process resembles the flow of a material fluid inside the syringe cylinder. Previous studies on 3D food printing have highlighted the importance of the rheological properties of the printed material, as these properties reflect the fluid flow characteristics and are considered key research elements [14].

During the printing process, food is constructed by depositing successive layers following a previously designed model. Furthermore, the careful selection of raw materials can contribute to improving both the quality and nutritional value of the produced food [15].

Different materials have been used for food printing, including protein, carbohydrates, or fat [13,16]. High molecular weight carbohydrates, like starch, are interesting since they undergo gelatinization (where water and heat break intermolecular bonds, allowing starch to bind more water) [16], producing a gel that is a suitable base for 3D ink formulations [17]. Moreover, starch can be obtained from abundant and inexpensive sources like potatoes. However, to make the formulation more nutritionally beneficial, it would be advantageous to enrich it with nutrient dense sources [13]. Currently, there is a growing interest in using more sustainable materials for 3D food printing, with a particular focus on using food waste. For example, incorporating grape pomace and broken wheat into cookie dough formulations has been explored as a 3D printing ink. These ingredients not only enhance the dough’s nutritional profile with added fiber and antioxidants but also contribute to the sustainable use of food byproducts [18]. Similarly, mushroom byproducts [19] have been used in the formulation of edible inks, and they have even improved the stability of the shape in pastes made of potato flour, thanks to the presence of proteins and fiber. Muthurajan et al. [20] used potato peel as an ingredient combined with wheat flour in the formulation of noodles prepared by 3D printing and observed that by incorporating potato peel powder, the noodles gain additional fiber, vitamins, and minerals.

In this context, carob pulp has been used in the 3D printing of chocolate as a sweetener [21]. The authors reported that samples containing 30% of carob pulp extract were scored with the most pleasant outcomes in sensory tests. It has also been used as flour for 3D printed cookies [22]. Their study found that carob pulp flour enhances 3D printed cookie formulations by adding nutritional value, specifically fiber, and positively impacting dough texture and stability. Carob flour also improved the final printed structure of cookies, allowing for precise shapes and better consistency. Research on carob pulp as an ingredient for 3D food printing formulations is limited, creating a promising area for study. The aim of this work is to evaluate the effect of the addition of a cellulose-rich fraction coming from carob pulp in the formulation of edible ink based on potato peel flour. The inks were characterized in terms of viscosity, water distribution, and printability. After printing, the pastes were evaluated in terms of textural parameters and porosity.

## 2. Results and Discussion

### 2.1. Characterization of the Carob Cellulose-Rich Fraction (CRF)

A yield of 44.5 ± 3.0 g CRF per 100 g of carob pulp was obtained, indicating that approximately 45% of the carob pulp can be considered dietary fiber. This value is closely aligned with the findings reported by Nasar-Abbas [23], who estimated the fiber content of carob pulp to be around 40%.

#### 2.1.1. Analysis of Carbohydrate Composition

Monosaccharides composition of the carob cellulose-rich fraction (CRF) is shown in Table 1. As shown, more than 65% of the CRF corresponds to the sugars present.

The total monosaccharides represent approximately 25% of the carob pulp. Table 1 also highlights a high glucose content, indicating significant amounts of cellulose (24.4%). Additionally, a high concentration of pectic polysaccharides, or pectins, is notable. This is evidenced by the detection of a considerable number of uronic acids, particularly galacturonic acid. Furthermore, although in smaller amounts compared to uronic acids, significant levels of rhamnose, arabinose, and galactose were found; these monosaccharides are components of the pectin structure [24]. Other sugars detected, such as xylose, mannose, and fucose, suggest the presence of small amounts of hemicelluloses, likely xyloglucans and xylans [25]. In addition to the polysaccharides that form the cell walls of carob pulp, significant amounts of lignin were also estimated. Specifically, for every gram of CRF, up to 340 mg of lignin was found.

#### 2.1.2. Functional Properties

The chemical structure of the polysaccharides that make up the plant cell wall is closely related to the functional properties of the cellulose-rich fraction (CRF). Table 2 presents the values of the functional properties determined for CRF (swelling capacity (Sw), fat adsorption capacity (FAC), and water retention capacity (WRC)).

The swelling capacity of the sample (CRF) was of the same order as that reported by Petkova et al. [26] (11.4 mL/g) for the galactomannan extract from carob flour, but it was considerably lower than the value reported by Fidan et al. [27] (30 mL/g) for the polysaccharide extract obtained from carob seeds. These differences may be attributed to the fact that swelling capacity can be influenced by climate and agricultural conditions. Additionally, it is known that extracts from different parts of the carob plant may exhibit different functional properties [26].

The fat adsorption capacity of carob CRF was lower than that obtained by Petkova et al. [26] for the galactomannan extract from carob flour. The FAC results were higher than the water retention capacity (WRC), suggesting that CRF could be suitable for use in the formulation of food emulsions.

The water retention capacity of carob CRF was higher than that of the galactomannan extract from carob flour, which was 0.14 g/g [26]. However, the value was very similar to that obtained for a polysaccharide extract from carob seeds (8.3 ± 0.02 g/g) [27] and to that of carob gum (5.6 g/g) [28]. This indicates that during the polysaccharide extraction process, a structural change occurs, making hydrophilic groups more accessible. The composition and structure of proteins and their interactions with other substances control the functional properties of carob CRF. WRC is essential for the sensory evaluation of formulated food products, as it represents the ability to absorb and retain hydrodynamic, capillary, and physically trapped water against gravity. WRC affects texture, juiciness, taste, and especially the shelf life of products.

### 2.2. Characterization of Cellulose-Based Gels

To evaluate the suitability of the different cellulose-based gels prepared as raw materials for the development of new 3D printed foods, various properties such as apparent viscosity, water distribution, and printability were assessed.

#### 2.2.1. Result of Apparent Viscosity

Apparent viscosity is a critical property influencing extrusion-based 3D printing. Its value must fall within a range low enough to allow the material to pass through the printer nozzle yet high enough to ensure proper adhesion between printed layers [29].

**Figure 1:**
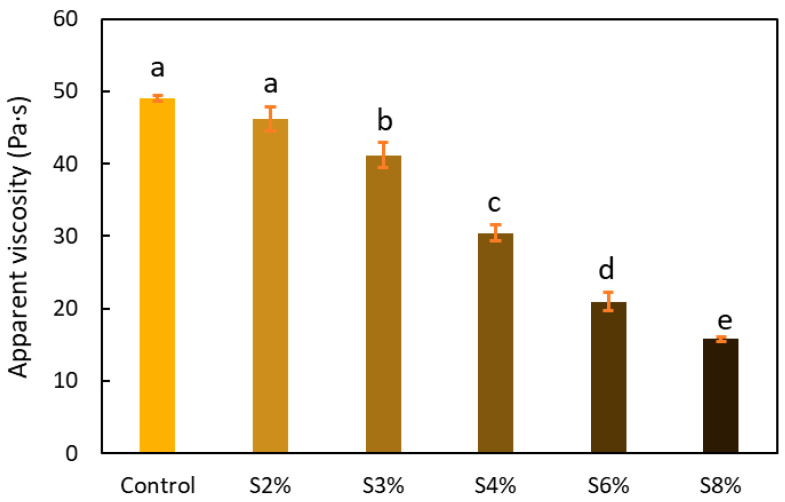
Apparent viscosity of the cellulose-based gels formulated with 0%, 2%, 3%, 4%, 6%, and 8% CRF. Different lowercase letters indicate significant differences (*p* < 0.05).

Figure 1 displays the apparent viscosity values of the different cellulose-based gels prepared, including the control (0% CRF) and the gels containing different proportions of CRF (2% to 8%). The addition of higher percentages of CRF caused a significant decrease in viscosity, with statistically significant differences observed between the gels containing carob fiber (*p* < 0.05). This can be attributed to the substitution of potato peel, the gelling agent due to its starch content, with carob fiber, as the total solids content (15%) remained constant across all experiments. No significant differences (*p* > 0.05) were observed at lower concentrations of added carob fiber, as seen between the control gel and the gel with 2% CRF. However, in the rest of the inks, significant differences (*p* < 0.05) were observed, even when the fiber increment between samples did not exceed 1 to 2%. This same effect was reported by Reche et al. [19] when potato flour was replaced by mushroom byproduct flour.

Inks for 3D printing should have adjusted and controllable viscosity—low under shear for smooth extrusion and high at rest to maintain stability after deposition. They need thixotropic behavior, reducing viscosity during extrusion and recovering it quickly after to retain structure. Adequate elasticity is essential to prevent collapse between printed layers, especially for complex builds. The ink must also be self-supporting to withstand its weight without deformation, requiring a balance of viscosity and elasticity [30].

#### 2.2.2. Result of Water Distribution

**Figure 2:**
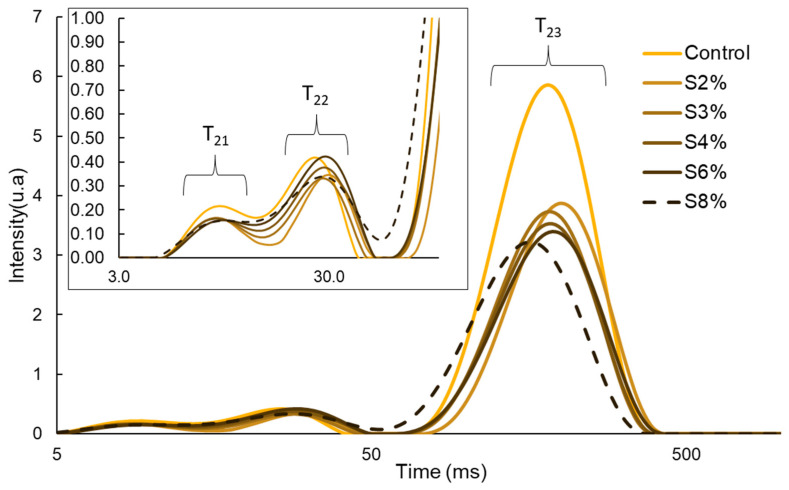
Distributions of the relaxation time after inverse Laplace transform analysis of the LF-NMR spectra of the cellulose-based gels formulated with 0%, 2%, 3%, 4%, 6%, and 8% CRF.

Water distribution is closely related to the properties and structure of the material and can be analyzed in cellulose-based gels for 3D printing using nuclear magnetic resonance (NMR) [31]. Figure 2 shows the relaxation time corresponding to each of the analyzed samples.

The transverse relaxation time (T₂), determined through the LF-NMR analysis, indicates the degree of water mobility within the cellulose-based gels. A lower T₂ value signifies that water is more tightly bound to the solid components, while a higher T₂ value suggests greater water mobility, as it is less bound [32].

Regarding the two peaks, T_21_ and T_22_, these correspond to water bound to different macromolecules. Moreover, the existence of two peaks indicates the presence of one fraction of water that is more strongly bound (bound water) and a second fraction with lower affinity (immobilized water). When analyzing the gels containing CRF in comparison to the control gel, it was observed that the control curve was shifted further to the left, indicating stronger cohesion of water molecules with the macromolecules formed by the various types of polysaccharides. In contrast, when evaluating only the CRF-containing gels, the area under the curve increased as the proportion of added CRF rose, up to a certain point where a significant decrease was noted, particularly in the S8% sample. This trend suggests that increasing CRF content enhances water retention in the gel. However, in the case of S8%, where the proportion of CRF exceeds that of the added potato peel, a distinct behavior was observed.

The T_23_ peak corresponds to longer relaxation times, representing free water within the gel matrix. It was observed that as the proportion of CRF increased, the peak shifted slightly to the left along the x-axis (indicating shorter times), suggesting that the mobility of the water is altered depending on the amount of added CRF. In the case of the S8% sample, a more pronounced shift towards shorter relaxation times was noted, which suggests a significant reduction in water mobility. Furthermore, the addition of CRF led to a decrease in both the intensity and area of the peaks, indicating a reduction in the amount of free water within the sample. These changes in the relaxation time of the T_23_ peak can be explained by the high water retention capacity of CRF within the gel, thereby reducing the free water content and increasing the stability of the resulting gel. This same effect was observed by Reche et al. [19] when replacing potato purée with mushroom flour.

#### 2.2.3. Result of Printability

The cellulose-based gel intended for 3D printing must possess an optimal composition that provides the best extrusion and shape retention capabilities [15]. As previously mentioned, the printability of each formulation was evaluated by printing the gels in a floral shape to assess geometric accuracy and stability. Table 3 presents representative images of the cellulose-based gels that were 3D printed, along with the printability rating for each formulation on a scale from 1 (very poor) to 5 (very good) [13].

The control samples did not receive the highest score due to the high viscosity of the gel, which hindered the extrusion, causing it to be unevenly distributed throughout the structure, resulting in a significant number of holes. Reche et al. [19] attributed this effect to the excessive starch content in potato peel, which impeded the pass of the gel through the nozzle and, consequently, led to structural imperfections characterized by the presence of holes. Regarding the S2% and S3% samples, the layers were clearly distinguishable, and the gear shape was printed with excellent definition. However, starting from S4%, the clarity of the shape and layer separation diminished. At higher proportions, particularly in the S8% sample, where the proportion of carob fiber exceeded that of potato peel, the separation between the applied layers was no longer distinguishable.

### 2.3. Characterization of 3D Printed Samples

#### 2.3.1. Result of Textural Properties

**Figure 3:**
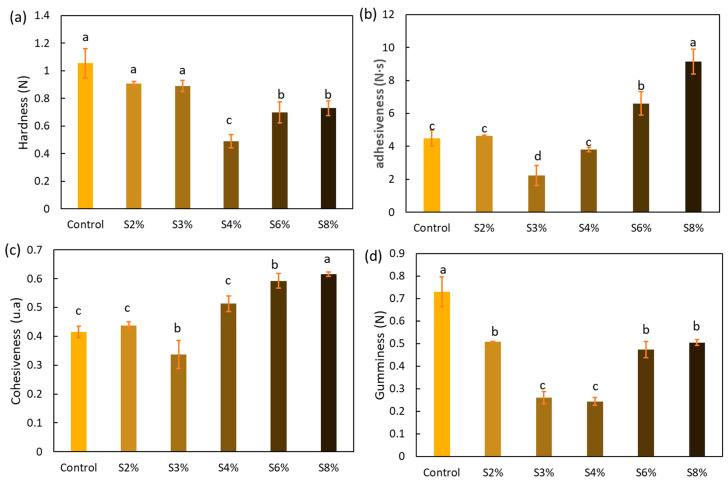
Textural properties: hardness (**a**), adhesiveness (**b**), cohesiveness (**c**), and gumminess (**d**) of the printed gels (shape-stable) formulated with 0%, 2%, 3%, 4%, 6%, and 8% CRF. Different lowercase letters indicate significant differences (*p* < 0.05).

Textural properties such as hardness, cohesiveness, adhesiveness, and gumminess are important characteristics of 3D printed foods [33]. Figure 3 shows the results of the textural properties, including hardness (a), adhesiveness (b), cohesiveness (c), and gumminess (d).

Hardness is related to the first bite sensation in the mouth and is indicated by the maximum force of the first compression cycle [34]. As shown in Figure 3a, the samples with 2% and 3% added CRF did not exhibit significant differences compared to the control sample (*p* > 0.05). On the other hand, the samples with a higher percentage of incorporated carob fiber (4%, 6%, and 8%) showed a significant decrease in hardness (*p* < 0.05), with the most pronounced reduction observed in the sample containing 4% added CRF. This could be related to the apparent viscosity and water distribution observations mentioned earlier. When a portion of the potato flour was replaced with carob fiber, the starch content decreased, leading to a significant reduction in the apparent viscosity of the ink and the water-binding strength. Consequently, this contributed to a decrease in hardness after printing, as starch is the key gelling component [19].

Adhesiveness can be defined as the negative area of force at the end of the first compression when the probe is retracted. It can be interpreted as a sticky sensation in the mouth [35]. As shown in Figure 3b, this property exhibits a significant increase when comparing the control gel or S2% with the S8% sample, the latter having the highest proportion of added CRF.

Cohesiveness refers to the strength of the internal bonds that make up the structure of a food, as well as how much a food can be deformed before it breaks. It is calculated as the ratio of the positive force area of the second compression to that of the first compression [36]. Regarding this property, the results are similar to those obtained for adhesiveness. As shown in Figure 3c, a slight decrease is observed at low proportions of added CRF, followed by a significant increase compared to the control gel (*p* < 0.05) when the CRF percentages rise to 8%.

Gumminess represents the energy required for food to reach a swallowable state and is defined as the product of hardness and cohesiveness [37]. This is a highly relevant textural characteristic, especially in semisolid foods with low hardness and high cohesiveness [34]. The results presented in Figure 3d were quite similar to those for hardness. Maximum gumminess was observed in the control gel, although significant increases in gumminess values were noted at high percentages of CRF.

#### 2.3.2. Microstructure

**Figure 4:**
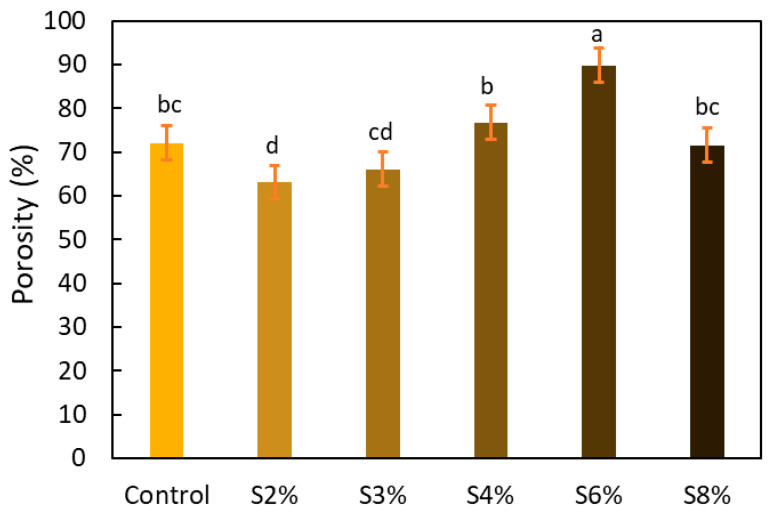
Percentages of porosity for the printed samples across the different formulations. Different lowercase letters indicate significant differences (*p* < 0.05).

Table 4 presents the images obtained via scanning electron microscopy (SEM) related to the printed samples; on the one hand, the control sample, and on the other, the prints made with gels incorporating CRF at concentrations of 2, 3, 4, 6, and 8%. The control sample exhibits an open structure with parts in a honeycomb pattern. The addition of CRF to the gel resulted in a closure of the structure; however, as the proportion of CRF increased, smaller cavities began to emerge. To corroborate the information obtained from the SEM images, the porosity of the different samples was measured. Figure 4 shows the percentages of porosity for the printed samples across the different formulations.

As shown in Figure 4, there is an upward trend in porosity values as the proportion of CRF increases, except in the case of the S8% sample, which exhibits a significant decrease (*p* < 0.05). Overall, an increase in porosity was observed among the control gel, S2%, S3%, S4%, and S6% samples, with particularly significant changes noted for the S6% sample compared to the control ink, indicating an increase in porosity. It is evident that the addition of CRF introduces openings within the microstructure of the inks, which is reflected in the increased porosity corresponding to the higher proportions of added CRF. In the case of S8%, the observed decrease is likely due to structural collapse, as the proportion of the gelling agent (starch derived from potato peel) has diminished to the point where the structure can no longer be maintained, resulting in a reduction in porosity values.

## 3. Conclusions

This study demonstrated the potential of carob residue as a valuable source of dietary fiber, with a high fiber content (44.5 ± 3.0%) and a cellulose content of 24.4%. Our findings are significant, as cellulose, a key component of dietary fiber, plays an important role in disease prevention. In addition to its composition, the functional properties of CRF (Carob Residue Fiber) are noteworthy. Each gram of CRF was capable of adsorbing approximately 6 g of lipids and retaining up to 4 g of water, highlighting its potential use as an ingredient in food formulations, particularly for developing cellulose-enriched products. The feasibility of using cellulose-based gels, derived from agro-industrial byproducts such as potato peel and carob pulp, for 3D printing of structured foods was confirmed. The addition of CRF to these gels significantly impacted their rheological, textural, and printability properties. Viscosity decreased with increasing CRF content, with the optimal range for 3D printing performance being between 3% and 4%. Higher CRF concentrations (6% and 8%) resulted in increased cohesiveness and adhesiveness compared to the control gel. Moreover, while low CRF additions (<6%) did not significantly alter the microstructure, higher proportions (>6%) led to substantial structural modifications.

In conclusion, the results of this study support the use of carob residue fiber as an effective ingredient for the formulation of cellulose-based gels for 3D food printing. This not only opens new possibilities for the development of fiber-rich foods but also provides a sustainable approach to the valorization of agro-industrial byproducts.

## 4. Materials and Methods

### 4.1. Raw Matter

The raw material used in this study was a mixture of flours from six different varieties of carob, specifically the *Bugadera*, *Fulla de raó*, *Valenciana*, *Rotja*, *Franco*, and *De la Mel* varieties. All the carob samples were collected from Llucmajor, Majora (Balearic Island, Spain). All samples were collected during the autumn of 2022.

**Figure 5:**
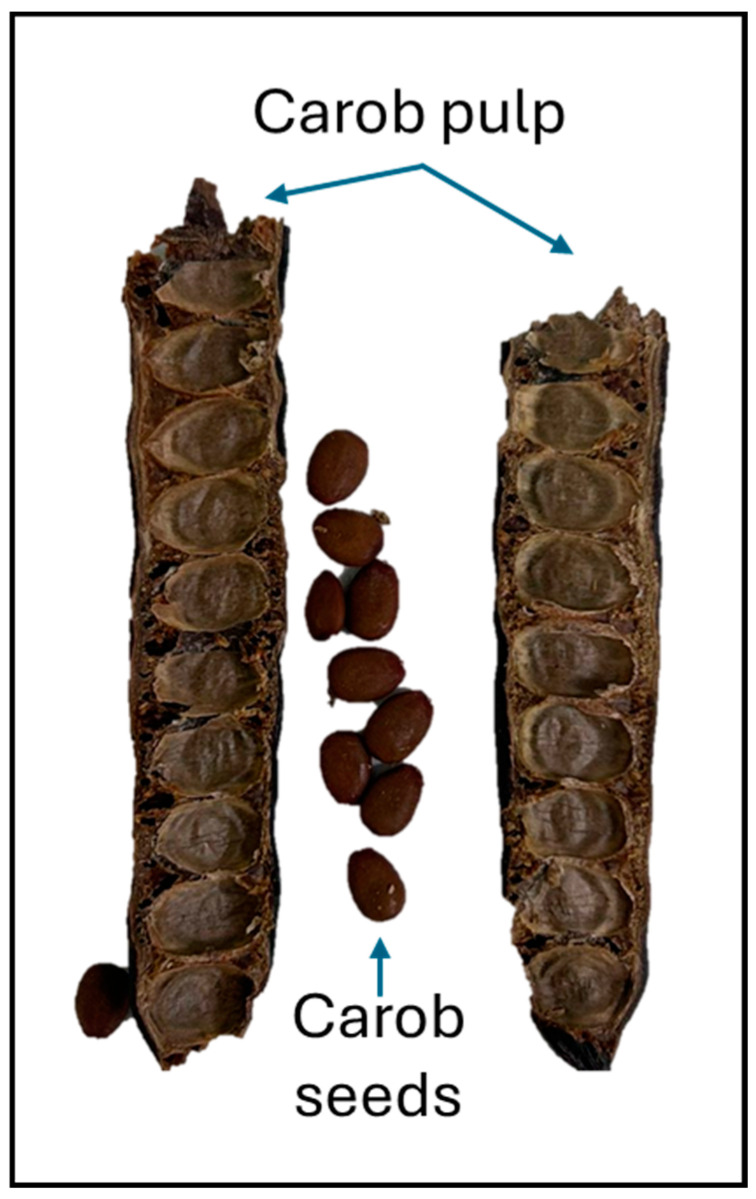
Photograph of the carob pulp and seeds of the *Bugadera* variety.

To obtain the flour mix, an equal amount (approximately 2 kg) of carob pulp (after removing the seeds) from each of the six varieties was ground with an IKA M20 laboratory mill (Barcelona, Spain). A photograph of *Bugadera* carob pulp and seeds can be observed in Figure 5 as an example. Subsequently, the flour obtained was sieved to obtain a particle size smaller than 0.25 mm.

Once the different flours were prepared, equal amounts of each were mixed to obtain a homogeneous blend of flours. From this flour, the cellulose-rich fraction of carob pulp was obtained (CRF).

### 4.2. Obtainment of the Cellulose-Rich-Fraction (CRF)

The CRF was extracted by obtaining the AIR (Alcohol Insoluble Residue). The procedure used to obtain the AIR has been previously described by Femenia et al. [24], with some modifications. Approximately 20 g of carob pulp flour was mixed with ethanol (1 L) with a concentration of 85% (*v*/*v*). The mixture was homogenized for 1 min at 13,000 rpm with an Ultra Turrax (Heidolph DIAX 600, Schwabach, Germany). Thereafter, the mixture was brought to a boil (about 80 °C) for 5 min to inactivate the enzymes that could cause the degradation of the various polysaccharides in the cell wall. Then, the sample was homogenized again for 2 min at 13,000 rpm and brought to a boil for 1 min. The resulting mixture was filtered using a glass fiber (Whatman GF-C, Maidstone, UK). Two additional washes were carried out in ethanol (about 100 mL), with the final one performed in absolute ethanol. The sample was then filtered and rinsed with absolute ethanol and acetone (about 100 mL each). The yield of AIRs was expressed in grams of AIR per 100 g of carob pulp flour. This material was in powder form.

### 4.3. Composition of the CRF

The neutral sugars that constitute the polysaccharides were released through acid hydrolysis (Saeman hydrolysis) in sulfuric acid and subsequently converted into alditol acetates. These monosaccharides (rhamnose, fucose, arabinose, xylose, mannose, galactose, and glucose) were then separated and analyzed using gas–liquid chromatography, following the method described by Dalmau et al. [38]. The uronic acids were quantified by colorimetry as total uronic acid from samples hydrolyzed for 1 h at 100 °C [39].

From the previously described analysis, cellulose was determined by multiplying the content in glucose by 0.9 [40].

### 4.4. CRF Functional Properties

In this study, the properties related to the hydration process were evaluated, specifically the swelling capacity (Sw) and water retention capacity (WRC), as well as the ability to absorb lipids, expressed as fat adsorption capacity (FAC).

For the Sw, approximately 1 g CRF (m_1_) was placed in a test tube containing 10 mL of a 1 M sodium phosphate buffer solution at pH 6.2, which simulates the pH of many foods. The initial volume of the sample was measured (V_1_). The sample and buffer were left to stand for 24 h to reach equilibrium, corresponding to the maximum swelling the sample could achieve (V_2_). After 24 h, the volume of the hydrated sample in the test tube was measured [41]. Sw was calculated according to Equation (1).

(1) $$Sw=\frac{V_{2}-V_{1}}{m_{1}}$$

The WRC was measured by suspending about 1 g (m_2_) of CRF in an excess of a 1 M sodium phosphate buffer solution (pH 6.2) for 24 h. After this time, the suspension was centrifuged (ALC 4218 centrifuge, Thermo Scientific, Milan, Italy) at 1750 g for 25 min. The two resulting phases, solid and liquid, were then separated by decantation, and the solid phase was weighed (m_3_). WRC was calculated as stated in Equation (2). Similarly, the FAC was measured by suspending the sample previously weighted (m_4_) of CRF in sunflower oil for 24 h. After this time, the solid and liquid phases were separated by centrifugation (25 min at 1750 g) and decantation. The solid phase was weighed (m_5_), and the FAC was calculated as stated in Equation (3) [42].

(2) $$WRC=\frac{m_{3}-m_{2}}{m_{2}}$$

(3) $$FAC=\frac{m_{5}-m_{4}}{m_{4}}$$

### 4.5. Preparation of the Cellulose-Based Gel for 3D Printing

Different cellulose-based gels were prepared using dry potato peel flour as the base of the formulation. The potato peel flour was obtained by drying the potato peels at 60 °C for 7 h in a convective oven (Dry Fruit Machine, Akcome Europe GmbH, Hangzhou, China). Thereafter, they were grounded and sieved (<0.5 mm). The CRF was used to partially replace potato peel flour in varying proportions, as shown in Table 5 while ensuring that the total solid content remained at 15% (wet matter) across all samples. A control gel was prepared using only potato peel flour. The CRF and the potato peel flour were weighed in powder form and mixed with distilled water previously heated to 90 ± 5 °C, with mechanical stirring (700 rpm), to allow the starch present in the potato peel flour to gelatinize, thus obtaining the appropriate consistency for printing, following the methodology described by Liu et al. [43,44]. Then, the gel was cooled in an ice water bath until reaching a temperature of about 20 ± 3 °C.

### 4.6. Characterization of the Cellulose-Based Gel

Different properties of the cellulose-based gel were determined to evaluate the effect of the addition of CRF.

#### 4.6.1. Apparent Viscosity

The apparent viscosity of the cellulose-based gel was measured using a VISCO R rotational viscometer (Selecta, Barcelona, Spain) equipped with a type R7 spindle of 3 mm in diameter, operating at a rotation speed of 50 rpm. The temperature during the analysis was kept constant at 21 ± 2 °C.

#### 4.6.2. Water Distribution

The water distribution within the cellulose-based gels was evaluated by low-field nuclear magnetic resonance using an Mq20 NMR analyzer (Bruker Biosciences, Madrid, Spain) with a magnetic field strength of 0.47 T, resulting in a frequency of 19.95 MHz, while the magnet temperature was maintained at 40.0 ± 0.1 °C. All samples were wrapped in plastic film and placed in 18 mm diameter tubes. The relaxation time (T_2_) was determined, as this parameter is related to the structure and rheological properties of the material, using Carr-Purcell-Meiboom-Gill (CPMG) sequences [45,46].

The T_2_ relaxation curve was mathematically modeled as an exponential function, and the inverse Laplace transform (ILT) was applied to obtain the relaxation time distributions from the exponential decay curve. This analysis was carried out using the Origin software (Origin 2021b, Northampton, MA, USA) following the methodology described by Sánchez-Alonso et al. [47].

#### 4.6.3. Printability

The cellulose-based gels, maintained at a temperature of 20 ± 3 °C, were transferred into printing syringes for the 3D printing process. A Foodini 3D printer (Natural Machines, Barcelona, Spain) was used. This device is a commercial extrusion-based printer with a 1.5 mm diameter cannula. Samples were printed in the form of two square prisms. The template list available in the Foodini printer software was selected, with a size of 2 × 2 × 1 cm and a total of seven layers for measuring textural properties, while two square prisms of 1.5 x 1.5 × 0.7 cm with a total of five layers were also printed to determine structural properties. Finally, a gear with a 4 cm radius and 0.7 cm height was printed with a total of five layers to determine printability. Each layer had a height of 1.4 mm and a printing speed of 1000 mm/min. The printing conditions were optimized beforehand through preliminary experiments.

Photographs of the structures were taken immediately after printing and were visually evaluated using a scale ranging from 1 (very poor) to 5 (very good), based on printing precision, smoothness of the printed products, and shape stability, according to the method described by Lille et al. [13].

### 4.7. Analysisof 3D Printed Samples

Different properties of the 3D printed samples were determined to evaluate the effect of the addition of CRF in cellulose-based gels.

#### 4.7.1. Textural Properties

Texture analysis was performed through mechanical tests with a ZWICK Z100 device (Zwick GmbH & Co., Ulm, Germany) equipped with a 200 N load cell and a force measurement accuracy of 0.20%. Each sample was subjected to two consecutive compression cycles until achieving a 40% deformation, which represents the action of two bites. All experiments were performed at room temperature. The objective was to collect data that would allow the calculation of different textural parameters such as hardness, cohesion, adhesiveness, and gumminess using the method described by Bourne et al. [48], applying the Origin software (Origin 2021b, Northampton, DT, USA). Three replicates of the experiments were performed on different printed samples, and the results were expressed as the mean ± standard deviation.

#### 4.7.2. Microstructure

The microstructure of the samples was evaluated using scanning electron microscopy (SEM) following the methodology described by Reche et al. [49], where the samples were subjected to a freeze-drying process using a LyoQuest device (Telstar, Barcelona, Spain). This process was performed at a pressure of 0.3 mbar and a temperature of −50 °C for 72 h. After the freeze-drying process, the samples were fractured using cryofracture with liquid nitrogen and fixed on support using carbon adhesive and were directly observed in a Hitachi (Tokyo, Japan) scanning electron microscope, model S-3400N, with a resolution of 5 μm, an acceleration voltage of 10 kV, and a working pressure of 40 Pa.

#### 4.7.3. Porosity

Porosity was evaluated according to the method proposed by Baniasadi et al. [50], where the freeze-dried samples, using the same previous procedure, were weighed initially (m_6_) and measured to determine their volume. Subsequently, they were submerged in absolute ethanol for 48 h. Finally, a second weighing was performed (m_7_), and porosity (Φ) was calculated using Equation (4).

(4) $$\Phi =\frac{m_{7}-m_{6}}{\rho *V}*100$$

where “ρ” corresponds to the density of absolute ethanol, and “V” corresponds to the volume of the sample.

### 4.8. Statistical Analysis

Statistical analyses were performed using R software version 4.2.2 [51] in conjunction with RStudio IDE [52]. The characteristics of the cellulose-based gels and printed pastes, such as textural properties, viscosity, and porosity, were measured in triplicate. Normality and homoscedasticity tests were performed for all studied variables using the Shapiro–Wilk test and the Levene test.

When the data followed a normal distribution and the variances showed homogeneity, ANOVA and Tukey tests were used to evaluate the existence and degree of significant differences, respectively. Significant variations were considered when the *p*-value was less than 0.05.

## Figures and Tables

**Table 1:** Monosaccharides composition of the carob cellulose-rich fraction (CRF).

Monosaccharid	mg/g CRF
Rhamnose	10.7 ± 1.6
Fucose	7.5 ± 1.1
Arabinose	78.4 ± 6.6
Xylose	158.2 ± 13.8
Mannose	24.8 ± 3.1
Galactose	47.4 ± 4.3
Glucose	179.0 ± 18.6
Uronic Acids	153.2 ± 9.0
Total	659.5 ± 42.2

**Table 2:** Functional properties of the carob cellulose-rich fraction (CRF).

Sw (mL/g)	FAC (g/g)	WRC (g/g)
5.9 ± 0.5	6.5 ± 0.5	4.0 ± 0.1

**Table 3:** Representative pictures of printed samples with different formulations and printability marks for each sample on a scale from 1 (very bad) to 5 (very good).

	Top View	Front View	Scale
Control	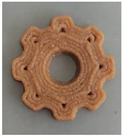	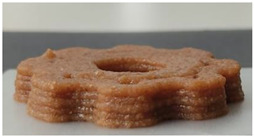	4
S2%	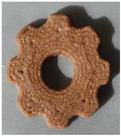	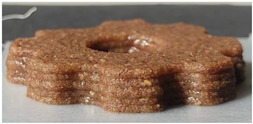	5
S3%	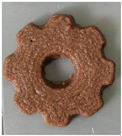	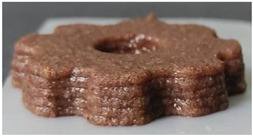	5
S4%	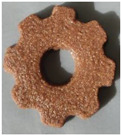	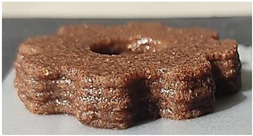	4
S6%	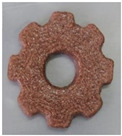	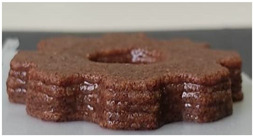	3
S8%	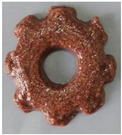	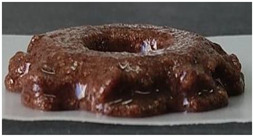	2

**Table 4:** Scanning electron microscopy (SEM) images of different formulations.

Control	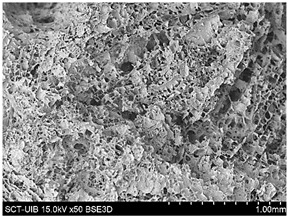	S2%	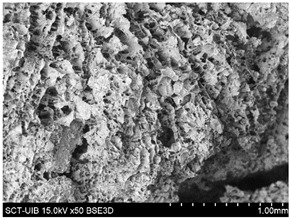
S3%	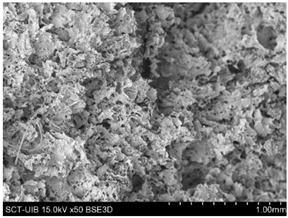	S4%	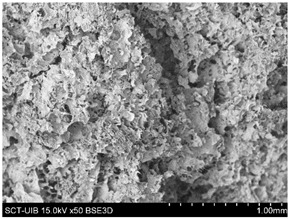
S6%	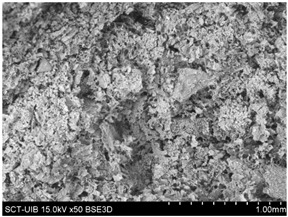	S8%	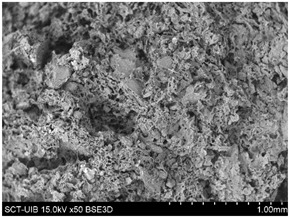

**Table 5:** Composition of the cellulose-based gels for the 3D printing.

Inks	CRF Content (%)	Potato Peel Flour (%)
**Control**	0	15
**S 2**	2	13
**S 3**	3	12
**S 4**	4	11
**S 6**	6	9
**S 8**	8	7

## Data Availability

The original contributions presented in this study are included in the article. Further inquiries can be directed to the corresponding author.
